# Immune Response, Viral Shedding Time, and Clinical Characterization in COVID-19 Patients With Gastrointestinal Symptoms

**DOI:** 10.3389/fmed.2021.593623

**Published:** 2021-06-17

**Authors:** Huan Yang, Xiangyu Xi, Weimin Wang, Bing Gu

**Affiliations:** ^1^Xuzhou Key Laboratory of Laboratory Diagnostics, School of Medical Technology, Xuzhou Medical University, Xuzhou, China; ^2^The First Affiliated Hospital of Suzhou University, Suzhou, China; ^3^Xuzhou Infectious Disease Hospital, Xuzhou, China; ^4^Laboratory Medicine, Guangdong Provincial People's Hospital, Guangdong Academy of Medical Sciences, Guangzhou, China

**Keywords:** COVID-19, gastrointestinal symptoms, immune response, clinical characterization, epidemiology

## Abstract

**Background and Aims:** Gastrointestinal (GI) symptoms are frequently observed in coronavirus disease (COVID-19) symptoms. Previous studies have mainly focused on epidemiology and characteristics in patients with GI symptoms, little is known about the roles of the immune response in susceptibility to and severity of infection. Here, we analyzed COVID-19 cases to determine immune response and clinical characteristics in COVID-19 patients with GI symptoms.

**Methods:** Based on the presence of GI symptoms, 79 patients in Xuzhou were divided into GI and non-GI groups. A retrospective study investigating the clinical characteristics, selected laboratory abnormalities, immune response, treatment, and clinical outcome was performed to compare patients with or without GI symptoms.

**Results:** Approximately 25% of patients reported at least one GI symptom. Our results showed significantly higher rates of fatigue, increased LDH, increased CK, higher percentage increase neutrophil-to-lymphocyte ratio (NLR), lymphopenia, and bilateral pneumonia in patients with GI symptoms. No significant changes in serum amylase (SAA), immunoglobulin (Ig) G, IgM, C-reactive protein (CRP), procalcitonin (PCT), interleukin-6 (IL-6), viral shedding time, liver injury, and kidney injury between the two groups were observed. The clinical type on admission of patients with GI symptoms reported significantly higher rates of critical disease type (20 vs. 3.3%; *p* = 0.033). However, the survival rate did not differ between the two groups.

**Conclusions:** Increase in total lymphocytes and NLR as well as the elevation of CRP, SAA, PCT, IL-6, CK, and LDH were closely associated with COVID-19 with GI symptoms, implying reliable indicators COVID-19 patients with GI symptoms were more likely to develop into a severe disease.

## Introduction

The coronavirus disease 2019 (COVID-19) outbreak started in December 2019 in China has spread sharply all over the world (1). Although COVID-19 presents most commonly with respiratory symptoms, early reports have described GI symptoms in patients diagnosed with COVID-19 (2). Clinical observations have shown that patients with COVID-19, especially severe patients, have significantly reduced lymphocyte counts and increased neutrophils, and are accompanied by a large accumulation of cytokines, indicating an imbalance in the immune system (3). The epidemiological, clinical dynamic profile among observed in patients with COVID-19 with GI symptoms has begun to emerge (4–6), but the characteristics of the immune response in COVID-19 patients with GI symptoms are still not clear.

An immune response in COVID-19 patients with GI symptoms is urgently needed to guide clinical diagnosis, treatment, infection control, and vaccine design. IgM antibodies are indicators of current or recent infections and the earliest signs after exposure to pathogens. IgG antibodies are the most common antibodies to the disease, indicating that the disease or past infection has recovered. Therefore, the testing of SARS-CoV-2 IgG and IgM antibodies not only helps to diagnose COVID-19, but also helps to assess the infection status. However, the production of antibodies and their protective effects on the prognosis of COVID-19 patients with GI symptoms need to be clarified.

Increasing clinical data have indicated the NLR as a powerful predictive and prognostic indicator for severe COVID-19 (7, 8). However, the NLR in COVID-19 patients with GI symptoms and its relation to disease status and outcome remains to be determined.

IL-6 is a typical pro-inflammatory factor that causes cytokine storms, and IL-6 blocking strategies have been successfully used in the treatment of various chronic inflammatory diseases (9). Therefore, we monitored serum IL-6 levels in patients with COVID-19 to better understand prognosis and to implement effective treatment. CRP, SAA, and procalcitonin (PCT) are usually early indicators of acute viral infection.

## Methods

### Data Collection

We enrolled a total of 79 patients with COVID-19 following the Declaration of Helsinki, which was confirmed by detecting SARS-CoV-2 RNA in throat swab samples using a SARS-CoV-2 nucleic acid detection kit according to the manufacturer's protocol (Shanghai BioGerm Medical Biotechnology Co.,Ltd). All patients were initially admitted to the Affiliated Hospital of Xuzhou Medical University and Xuzhou Infectious Disease Hospital from 26 January to 16 February, 2020.

A retrospective study investigating patients age, sex, medical history, symptoms, comorbid conditions, severity assessment on admission, laboratory findings, immune response parameter, chest CT findings, treatment, efficacy, and clinically-relevant hospitalization outcomes was performed.

The definition of positive GI symptoms requires patients to present at least one of the following symptoms: nausea, vomiting, or diarrhea. GI symptoms are recorded on admission, to exclude the effects of other drug treatments and external factors. COVID-19 patients are divided into four subtypes according to their symptoms. The severity of the disease is determined according to the diagnosis and treatment plan of SARS-CoV-2 in China (Sixth Edition). Mild patients have very mild symptoms, and imaging does not show signs of pneumonia. The common type has fever and respiratory symptoms, while imaging shows evidence of pneumonia. Severe patients present obvious clinical symptoms, shortness of breath and other symptoms, and changes in clinical indicators. The pulse oxygen saturation is <93% in the resting state, and the lung imaging progressed significantly within 24–48 h, reaching a degree of 50%. Critical patients have respiratory failure, require mechanical ventilation or non-invasive ventilation, or present shock or other organ failure, and require ICU monitoring and treatment.

### Laboratory Examination of Blood Samples

Approximately 3–5 mL of peripheral blood was obtained with a collection tube from the subjects in each group, serum samples were separated at 2,000 rpm for 20 min centrifugation. Serum cytokines were tested using enzyme-linked immunosorbent assay (ELISA). CRP was tested using i-CHROMA immunofluorescence assay. IgG and IgM antibodies were tested using quantum dot fluorescence immunoassay technology. PCT were tested by chemiluminescence analysis.

### COVID-19 Treatment

There is currently no targeted or specific treatment for COVID-19. We have attempted many treatment approaches, including antiviral therapy, antibiotics, steroid hormones, oxygen therapy, Traditional Chinese medicine treatment, probiotics, immunoglobulin treatment. For antibiotic treatment, we have attempted one or more antibiotics, which included moxifloxacin hydrochloride tablets, levofloxacin hydrochloride, ceftriaxone, cefoperazone, biapenem, daptomycin, and linezolid. We used lopinavir, ritonavir, and interferon-α as antiviral treatments. Probiotics were used to regulate the gut microbe and thus, we used *Bacillus* subtilis dual live bacteria capsules to relieve GI symptoms. Traditional Chinese medicine (TCM) treatment can regulate immunity. Lianhua Qingwen granules, Huoxiang Zhengqi capsules, and Chinese herbal decoctions. Immune globulin was used to treat patients at a dose of 20 g/day. Glucocorticoids such as methylprednisolone and inhaled budesonide were not used in patients with non-severe COVID-19 patients.

### Statistical Analysis

Variables are described by frequency and percentages, compared using the chi-squared (χ2) or Fisher exact tests. Normality of distribution was analyzed by Shapiro-Wilk test. When data had a normal distribution, statistical significance of differences between groups was calculated, followed by unpaired *t*-test and non-parametric test when appropriate. All statistical analyses were performed using SPSS version 23.0 software. *P* ≤ 0.05 was considered statistically significant.

## Results

A total of 79 patients with confirmed COVID-19 were enrolled from 26 January 2020 to 16 February 2020 in Xuzhou, among which 20 (25%) patients presented at least one GI tract symptom GI symptoms were as follows: 14 patients experienced diarrhea six patients had vomiting symptoms. Of the 20 COVID-19 patients with GI symptoms, eight were males and 12 females, with a mean age of 46.7 years (range 26–69). As outlined in Table 1, there were no age and sex differences between In addition, COVID-19 patients with GI symptoms were not significantly correlated with any medical history, including hypertension, diabetes or cancer (Table 1). Five (25%) patients had a history of exposure to Wuhan three (15%) patients had a history of contact with patients with COVID-19 and nine (45%) patients were exposed to a positive family cluster (Table 1). The proportion of critical disease type was significantly higher in patients with COVID-19 with GI symptoms than in those without GI symptoms (20 vs. 3.3%, *p* = 0.033).

**Table 1:** Epidemiological characteristics of patients with COVID-19 with and without GI symptoms.

**Characteristic**	**Group A:**	**Group B:**	***P*-value**
	**GI symptoms**	**No GI symptoms**	
	**(*n =* 20)**	**(*n =* 59)**	
Age (≥45 years old)	11 (55%)	24 (40.6%)	0.265
Sex (male)	8 (40%)	27 (45.7%)	0.654
Sex (female)	12 (60%)	32 (54.2%)	0.654
**Past medical history**			
Any	8 (40%)	29 (49.1%)	0.478
Hypertension	5 (25%)	10 (16.9%)	0.643
Diabetes	2 (10%)	8 (13.5%)	0.980
Cancer	1 (5%)	1 (1.7%)	0.445
Hyperthyresis	0	1 (1.7%)	>0.999
Coronary heart disease	0	3 (5%)	0.567
Cerebrovascular disease	0	4 (6.7%)	0.545
Pregnancy	0	1 (1.7%)	>0.999
**Exposure history**			
Contact with the epidemic area	5 (25%)	10 (16.9%)	0.643
Contact with patients	3 (15%)	30 (50.8%)	0.005
Family cluster	9 (45%)	17 (28.8%)	0.183
**Clinical subtype on admission**			
Critical	4 (20%)	2 (3.3%)	0.033
Coventional	16 (80%)	54 (91.5%)	0.32
Light	0	3 (5%)	0.567

As outlined in Table 2, clinical symptoms such as fatigue (65 vs. 23.7%; *p* = 0.001) were also more frequent in patients with GI symptoms than in those without GI symptoms. Although there were no statistical differences, COVID-19 patients with GI symptoms had higher rate of fever >38.5°C, sore throat and muscle ache. The percentage of patients with higher CK (25 vs. 1.7%; *p* = 0.004) and LDH (45 vs. 20.3%; *p* = 0.031) was significantly higher in COVID-19 patients with GI symptoms than in those without GI symptoms. However, other abnormal laboratory findings revealed no significant differences in the rate of hyperglycemia, coagulopathy, anemia, liver injury, and kidney injury between patients with GI symptoms and those without. Additionally, Chest CT findings showed that patients with GI symptoms had significantly higher rates of bilateral pneumonia (90 vs. 61%; *p* = 0.016).

**Table 2:** Clinical characteristics and selected laboratory abnormalities of patients with COVID-19 with and without GI symptoms.

**Characteristic**	**Group A:**	**Group B:**	***P*-value**
	**GI symptoms**	**No GI symptoms**	
	**(*n =* 20)**	**(*n =* 59)**	
Fever >38.5°C	9 (45%)	15 (25.4%)	0.100
Sore throat	6 (30%)	6 (10.1%)	0.076
Muscle ache	3 (15%)	1 (1.7%)	0.079
Headache	4 (20%)	5 (8.5%)	0.320
Fatigue	13 (65%)	14 (23.7%)	0.001
Chills	1 (5%)	5 (8.5%)	0.985
Dyspnea	10 (50%)	20 (33.8%)	0.200
Cough	14 (70%)	40 (67.7%)	0.855
Sputum production	9 (45%)	23 (38.9%)	0.636
Co-infection (*Enterobacter aerogenes, Enterococcus gallinarum*, Urinary Tract Infection)	0	5 (8.5%)	0.416
**Selected abnormal laboratory findings**			
Hyperglycemia (3.8–6.2 mmol/L)	11 (55%)	31 (52.5%)	0.849
Coagulopathy (PT 9.4–12.5 s, D-dimer 0–2.4 μg/mL)	4 (20%)	25 (42.3%)	0.073
Anemia (Hb115–150 g/L)	1 (5%)	6 (10.1%)	0.804
CK increased (20–200 U/L)	5 (25%)	1 (1.7%)	0.004
LDH inceased (110–240 U/L)	9 (45%)	12 (20.3%)	0.031
Liver injury (ALT 9–50 U/L, AST 15–40 U/L)	1 (5%)	5 (8.5%)	0.985
Kidney injury (Urea nitrogen 1.7–8.3 mmol/L, Creatinine 40–97 umol/L)	2 (10%)	2 (3.4%)	0.565
**Chest X—ray/CT findings**			
Normal	0	3 (5%)	0.567
Unilateral pneumonia	2 (10%)	15 (25.4%)	0.256
Bilateral pneumonia	18 (90%)	36 (61%)	0.0160
Multiple mottling and ground- glass opacity	18 (90%)	48 (81.3%)	0.581

**Figure 1:**
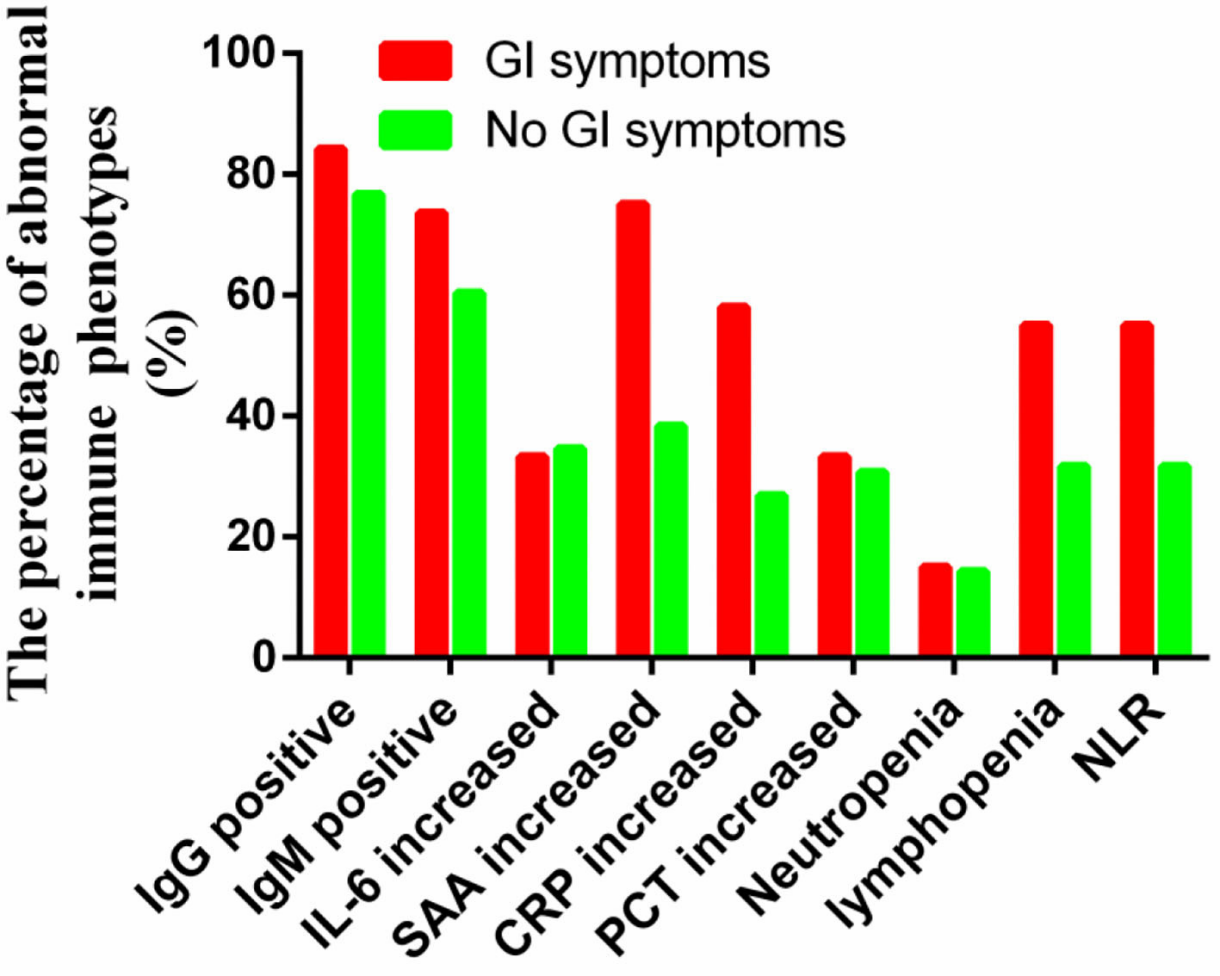
Immune response phenotype in COVID-19 patients with and without GI symptoms according to the percentage of abnormal indicators.

**Figure 2:**
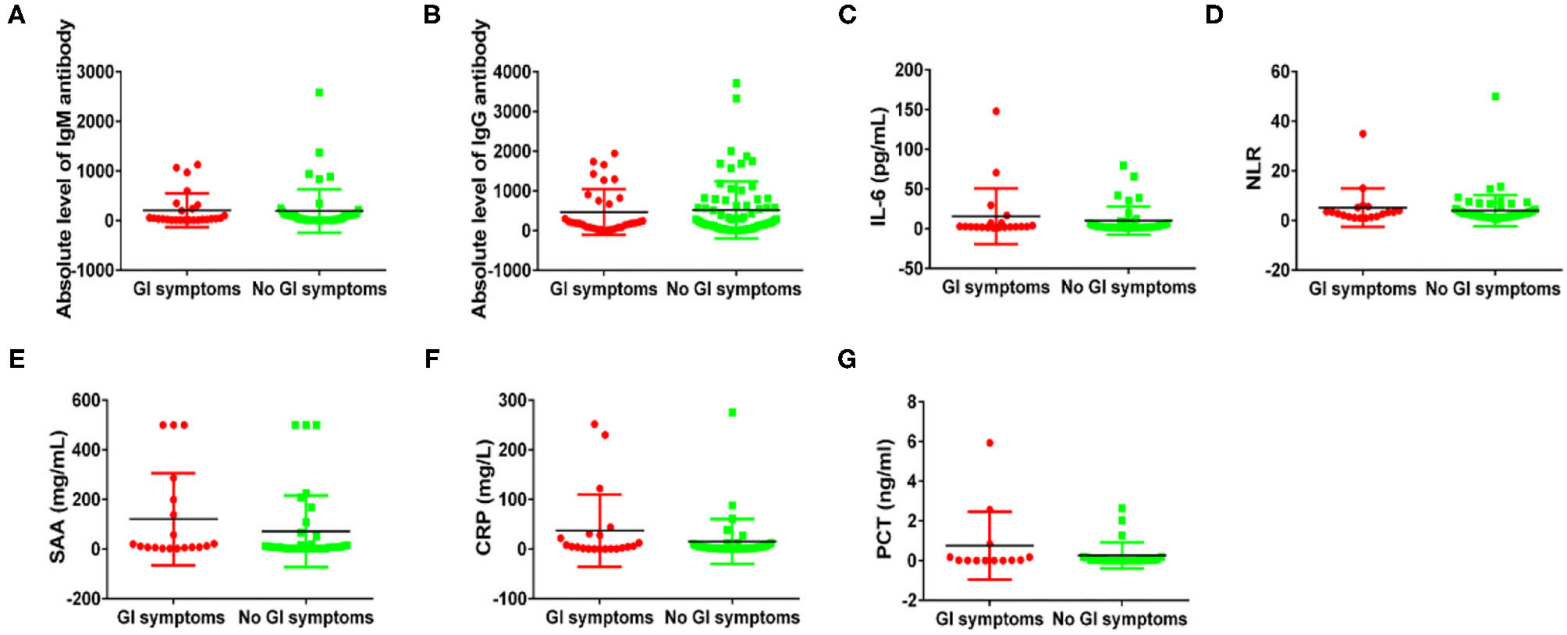
Immune response phenotyping in COVID-19 patients with and without GI symptoms according to the expression level. Comparing IgM levels **(A)**, IgG levels **(B)**, IL-6 levels **(C)**, NLR levels **(D)**, SAA levels **(E)**, CRP levels **(F)**, and PCT levels **(G)** between patients with and without GI symptoms.

Next, to compare the immune response induced by SARS-CoV-2 in COVID-19 patients between with GI symptoms and in those without GI symptoms, we investigated the protein expression of pro-inflammatory cytokines IL-6, the SARS-CoV-2 IgM and IgG antibody, lymphocyte, NLR (cut off 3.04), CRP, SAA, and PCT. The percentages of increased NLR (55.5 vs. 31.7%; *p* = 0.049), lymphopenia (55.5 vs. 31.7%; *p* = 0.049), increased SAA (75 vs. 38.5%; *p* = 0.059), increased CRP (58.3 vs. 26.9%; *p* = 0.061), and increased IgG antibody (84.2 vs. 76.7%; *p* = 0.743) and IgM antibody (73.6 vs. 60.4%; *p* = 0.316) were higher in COVID-19 patients with GI symptoms than in those without GI symptoms, although some test results showed no significant differences. However, the SARS-CoV-2 IgM, IL-6, and PCT were not differences between patients with GI symptoms and those without (Figure 1). We further analyzed the absolute expression level of IL-6, and the SARS-CoV-2 IgM and IgG antibodies, NLR, CRP, SAA, and PCT (Figure 2). Among COVID-19 patients with GI symptoms, the median level of IgM, IgG, IL-6, SAA, CRP, PCT, and NLR were 209.15 IU/mL, 470.36 IU/mL, 15.7 pg/mL, 120.6 mg/mL, 37.1 mg/mL, 0.76 ng/mL, and 8.76, respectively. In addition, for COVID-19 patients without GI symptoms, the median levels of IgM, IgG, IL-6, SAA, CRP, PCT, and NLR were 195.1 IU/mL, 521.1 IU/mL, 10.1 pg/mL, 71.8 mg/mL, 15.4 mg/mL, 0.268 ng/mL, and 6.35, respectively. Except for IgG, there was a similar tendency for a stronger immune response in COVID-19 patients with GI symptoms than in those without GI symptoms, although there were no statistically significant differences between patients with GI symptoms and those without. In sum, COVID-19 patients with GI symptoms experienced a more severe immune system disorder.

**Figure 3:**
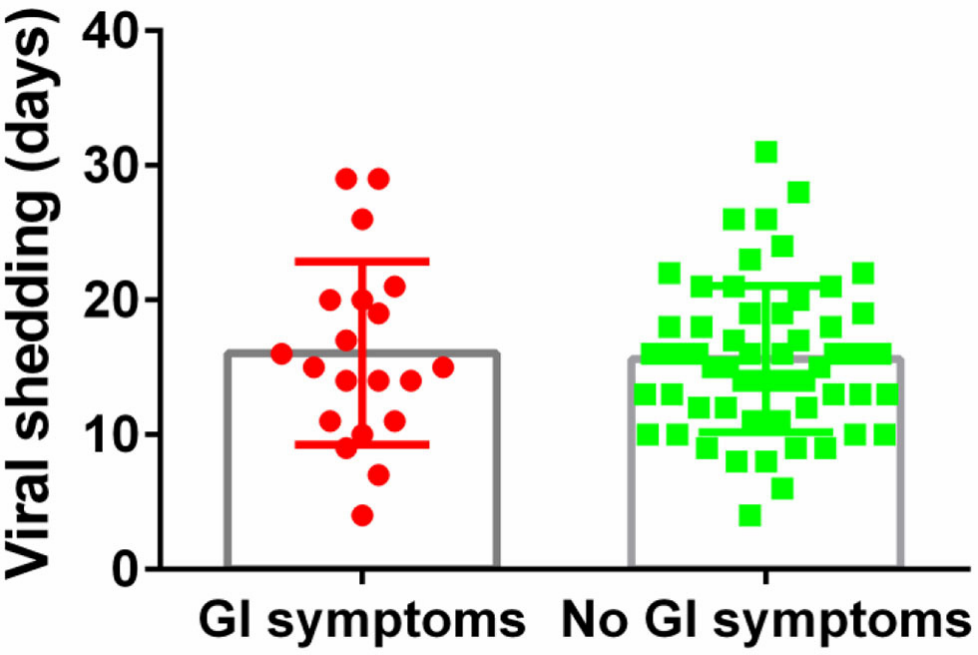
Viral shedding days between patients with and without GI symptoms.

The median duration of viral shedding was similar between patients with GI symptoms and without (Figure 3). For COVID-19 with GI symptoms patients, the median duration of viral shedding was 16.1 days, and the median duration of viral shedding was 15.6 days in patients without GI symptoms.

COVID-19 patients with GI symptoms receiving oxygen, antibiotics, antivirals, immunoglobulins, hormones, TCM and probiotics treatment accounted for 95, 90, 100, 10, 45, 60%, and 0, respectively (Table 3). No significant differences in rates of treatment with oxygen, antibiotics, antivirals, immunoglobulins, TCM and probiotics treatment, except for hormone treatment (45 vs. 19%; *p* = 0.031). At the end of observation, all patients were discharged.

**Table 3:** Treatment and Clinical outcome in patients with COVID-19 with and without GI symptoms.

**Variable**	**GI symptoms**	**No GI symptoms**	***P*-value**
	**(*n =* 20)**	**(*n =* 59)**	
**Treatment**			
Antiviral therapy	20 (100%)	59 (100%)	>0.999
Antibiotic treatment	18 (90%)	41 (69.5%)	0.068
Hormones	9 (45%)	12 (19%)	0.031
Oxygen therapy	19 (95%)	58 (98.3%)	0.445
Traditional Chinese medicine treatment	12 (60%)	22 (37.2%)	0.076
Probiotics treatment	0	3 (5%)	0.567
Immunoglobulin treatment	2 (10%)	1 (1.7%)	0.156
**Clinical outcome**			
Discharge from hospital	20 (100%)	59 (100%)	>0.999
Staying in hospital	0	0	
Death	0	0	

## Discussion

In this study involving two tertiary hospitals in Xuzhou, we found that nearly 25% of hospitalized patients with SARS-CoV-2 infection presented at least one GI symptom. Vomiting and diarrhea were the most common symptoms in these patients. Patients diagnosed with COVID-19 may only have GI symptoms. Therefore, in order to control the epidemic, it is necessary to rigorously screen patients and take appropriate treatment measures. Patients diagnosed with COVID-19 with GI symptoms patients, may be misdiagnosed which favors diffusion of the virus. These close contacts will not be isolated, and may lead to widespread spread of the virus and improper treatment of diagnosed patients. Patients suspected of COVID-19 presenting with GI symptoms, should be carefully managed as the ability of the virus bind to ACE2 receptor of the GI tract has been identified (10). Patients with GI symptoms have reported greater fatigue. The percentage of increased CK and LDH was significantly higher in patients presenting GI symptoms. LDH levels have been positively associated with the COVID-19 severity (11). Increased CK and LDH are indicators of heart damage, therefore, patients with GI symptoms should be monitored for heart disease. Patients with GI symptoms on admission are associated with the clinical types indicating significantly higher rates of critical type disease. Therefore, patients with GI symptoms should receive greater attention for the development of critical disease. It was consistent with other report that patients with COVID-19 with GI symptoms had significantly higher critical types than those without GI symptoms (6).

Lymphocytes and specific lymphocyte subsets played an important role in the maintenance of the immune system function. Lymphocytes and the subsets alterations were associated with the clinical characteristics and treatment efficacy of COVID-19, and critically ill patients showed a significant reduction in levels of lymphocytes, monocytes, CD4 + T cells, CD8 + T cells, CD3 cells, CD19 cells, and natural killer (NK) cells (12–14). The association between lymphopenia and the severity of COVID-19 implied as the depletion of lymphocytes with had occurred, especially CD4+T and CD8+T cells. Thus, it is important to clarify the different characteristics of lymphocytes in COVID-19 patients with GI symptoms and without. However, this study observed levels of total lymphocytes and did not detect its subsets. We found that lymphopenia was significantly higher in COVID-19 patients with GI symptoms than in those without, indicating immune system disorders during the course of SARS-CoV-2 infection with GI symptoms. This was not consistent with the findings by Luo et al. who reported that lymphocyte counts in COVID-19 patients with GI symptoms were similar in those without GI symptoms in Wuhan, China (14). For reasons that are not entirely clear, these differences may be related to the region and the number of patients. The high NLR is a reliable indicator for predicting the incidence of early more severe disease (15). Our results showed that patients with GI symptoms had higher levels of NLR than those without GI symptoms, suggesting that COVID-19 patients with GI symptoms and high NLR levels were more likely to develop into severe patients. In total, our study demonstrated the association between GI symptoms and lymphopenia, and provided important insights on the immunopathogenesis of SARS-CoV-2 infection with GI symptoms.

The SARS-CoV-2 specific IgM and IgG antibodies in patients provided the basis for disease diagnosis. In addition, patients with severe disease frequently presented a stronger IgG response and a higher titer of total antibodies, which was associated with worse outcome (7). However, some studies have reported that there was no direct correlation between antibody concentration and disease severity (13). Therefore, whether SARS-CoV-2 specific IgM and IgG antibodies in patients was pathogenic or protective required further study. Our study showed that there was no difference between COVID-19 patients with GI symptoms and those without IgM and IgG antibody levels. The IgM and IgG antibody responses in COVID- 19 patients, still require elucidation.

Infammatory markers such as CRP and PCT were associated with the COVID-19 severity. It has been reported that the levels of CRP were significantly increased in severe cases compared to non-severe COVID-19 patients (16). SAA can improve inflammation by activating chemokines and inducing chemotaxis (17), and were significantly related to COVID-19 severity. The PCT concentration of severe patients was significantly higher than that of non-severe patients (18). Although there was no significant difference, the percentage of increased SAA, increased PCT, and increased CRP and their concentrations were higher in COVID-19 patients with GI symptoms, which indicated patients with GI symptoms presented more intense inflammation. In the fight against SARS-CoV-2, excessive secretion of IL-6 could cause acute and severe systemic inflammatory cytokine storms (19). Although no clear correlation has been found between COVID-19 patients with GI symptoms and IL-6 levels, IL-6 may become a potential target for immunotherapy for COVID-19 pneumonia.

In our study, the SARS-CoV-2 viral duration in COVID-19 patients with GI symptoms and without was similar, which means they were equally effective in spreading COVID-19. For COVID-19 patients with only GI symptoms, nucleic acid testing for virus is the gold standard for diagnosis. According to the latest version of the Chinese guidelines (20), after discharge from the hospital, patients should remain isolated and receive close medical observation by doctors for an additional 14 days. Our study showed that the average viral shedding time for the COVID-19 patients with GI symptoms was 16 days, so medical observation require longer periods.

It should be noted that there were some limitations of this study. First, our study is was limited by its small sample size, thereby it is not known whether our findings could be generalized. Nevertheless, our cohort is representative of two tertiary care hospitals containing different COVID-19 diseases spectrums. Furthermore, the quantitative viral load was not available for our patients, so the kinetics of viral shedding and the magnitude of antibody response during COVID-19 disease progression remained unknown in this study. Peripheral lymphocyte subset alteration showed an obvious association with the clinical characteristics of COVID-19, our study did not monitor lymphocyte subsets, including CD4+T cells, CD8+T cells and B cells. Although multiple immune indicators were detected, there was no continuous monitoring of dynamic immune responses.

In conclusion, we reported, the immune response, viral shedding time and clinical characterization in of COVID-19 patients with GI symptoms. Although we did not find a correlation between the presence of GI symptoms and hospitalization outcomes, we noted that the rate of critical disease type was increased in COVID-19 patients with GI symptoms. The percentage of increased CK and LDH was significantly higher in COVID-19 patients with GI symptoms than in those without GI symptoms. Fortunately, COVID-19 patients with GI symptoms did not tend to present more serious liver or kidney damage. In addition, COVID-19 patients with GI symptoms showed more intense immune response, including increased NLR, lymphopenia, increased SAA and increased CRP levels, although there was no significant difference in the levels of specific IgG or IgM antibodies. Based on the characteristics of the immune response of patients with GI symptoms, it is suggested that immunotherapy such as antagonistic cytokines may be used to treat COVID-19 patients. Further study on the immunopathogenesis of SARS- CoV-2 infection with GI symptoms is warranted. Immunotherapy and other TCM, probiotics, antiviral, anti-inflammatory, antioxidant, oxygen inhalation, respiratory support, and protective effects of organ failure may be available, cost-effective, and of tolerable toxicity.

## Data Availability Statement

The original contributions presented in the study are included in the article/supplementary material, further inquiries can be directed to the corresponding author/s.

## Ethics Statement

Written informed consent was obtained from the individual(s), and minor(s)' legal guardian/next of kin, for the publication of any potentially identifiable images or data included in this article.

## Author Contributions

HY analyzed the data and drafted the paper. XX processed and collected data. WW and BG revised and proofreaded articles. All authors contributed to the article and approved the submitted version.

## Conflict of Interest

The authors declare that the research was conducted in the absence of any commercial or financial relationships that could be construed as a potential conflict of interest.
